# Myiase nasale à *lucilia* sp. chez un patient intubé : à propos d'un cas au Maroc

**DOI:** 10.48327/mtsi.v2i3.2022.233

**Published:** 2022-08-26

**Authors:** Manal BOUIKHIF, Yasmine EL KETTANI, Mohamed LYAGOUBI, Sarra AOUFI

**Affiliations:** 1 Laboratoire central de parasitologie et mycologie, Centre hospitalier universitaire Ibn Sina, Rabat, Maroc; 2 Faculté de médecine et de pharmacie, Université Mohamed V, Impasse Souissi, 10100 Rabat, Maroc

**Keywords:** Unité de soins intensifs, Infection, Myiase nasale, *Lucilia* sp., Hôpital, Rabat, Maroc, Maghreb, Afrique du Nord, Intensive care unit, Infestation, Nasal myiasis, *Lucilia* sp., Hospital, Rabat, Morocco, Maghreb, Northern Africa

## Abstract

**Introduction:**

Les myiases sont des infections parasitaires accidentelles ou obligatoires des animaux et de l'homme par les larves de mouches ou asticots. Les myiases en milieu hospitalier restent exceptionnelles.

**Observation:**

Un patient de 72 ans a été admis au service de réanimation pour la prise en charge d'un choc septique compliquant une péritonite secondaire. Le patient a été intubé. Quelques jours après, des larves mobiles de couleur blanche ont été découvertes dans les cavités nasales du patient et ont été adressées au laboratoire de parasitologie pour identification. L’étude microscopique des larves a permis de diagnostiquer une myiase à *Lucilia* sp.

**Discussion/Conclusion:**

Les myiases des unités de soins intensifs restent exceptionnelles et touchent préférablement les patients intubés ou présentant des lésions infectées ou nécrosées. Le traitement est l'extirpation en totalité des larves. La prévention de cette infection repose sur tous les moyens physiques et chimiques permettant d’éliminer les mouches dans le milieu hospitalier.

## Abstract (en)

**Introduction:**

Myiasis is the infestation of living bodies of animals and humans with fly larvae or maggots. These accidental or obligatory parasites grow in three stages in the host while feeding on his tissues. Myiasis in a hospital area is a rare situation when flies accidentally lay their eggs on patients, mostly in open wounds or cavities. Case presentation. We report a case of a 72-year-old patient admitted to a general surgery department for peritonitis, then to an intensive care unit (ICU) for septic shock where he was intubated. Some maggots were seen moving in his nasal cavity a few days after his admission and were sent to our laboratory for examination. The larvae were identified as the third larval stage of *Lucilia* sp. They were removed entirely after intense nasal washing. A checking examination of his nasal cavities revealed no evidence of further infestation. After nasal washing, no other larvae were seen but unfortunately, the patient died of septic shock a few days later.

**Discussion:**

Few cases of nasal myiasis in hospitalized patients were reported. ICU myiasis, extremely rare, is caused by the patient's inability to fend off the flies because of their conscious state, presence of blood or odors caused by infections, and the warm humid environment.

**Conclusion:**

Myiasis in intensive care unit is a delicate situation that might be underreported. Preventing this infection is directly related to the control of the flies.

## Introduction

La myiase est l'infection des animaux et de l'homme par les larves de mouches. C'est une infection rare qui intéresse les zones tropicales et subtropicales. Les myiases sont fréquentes dans les régions rurales pauvres sous-médicalisées avec de mauvaises conditions d'hygiène. Cette infection touche souvent la peau, les plaies, les yeux et les cavités. Au Maroc, la famille de mouches la plus incriminée dans les myiases est celle des Oestridae. Toutefois, quelques cas de myiase à *Lucilia* (famille des Calliphoridae) ont été rapportés. Nous décrivons un cas de myiase nasale due à *Lucilia* sp. chez un patient en sédation et intubé.

## Description du cas Clinique

Un patient de 72 ans a été admis au service de chirurgie viscérale pour la prise en charge en urgence d'une péritonite par perforation d'une hernie intestinale étranglée. Le patient a été opéré et a reçu une antibiothérapie à large spectre pendant quelques jours avant l'installation d'un choc septique par péritonite secondaire, pour lequel il a été transféré au service de réanimation. Le patient a été mis en sédation, intubé et ventilé et a été repris chirurgicalement pour sa péritonite post-opératoire. Sept jours après, lors des soins quotidiens, l'infirmière a constaté la présence de plusieurs larves mobiles et blanches dans les cavités nasales du patient. Ces larves ont été retirées à la main et ont été adressées au laboratoire de parasitologie pour identification. L'examen des cavités nasales n'a pas montré de lésions des muqueuses. Il n'existait pas d'autres larves dans les autres cavités ni sur les plaies chirurgicales du patient.

**Figure 1:**
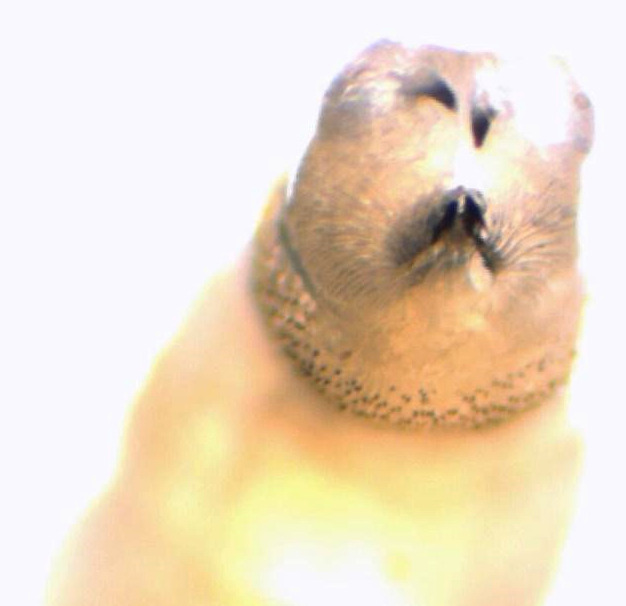
Deux crochets visibles à l'extrémité antérieure pointue de la larve Two hooks visible at the pointed anterior end of the larva

**Figure 2:**
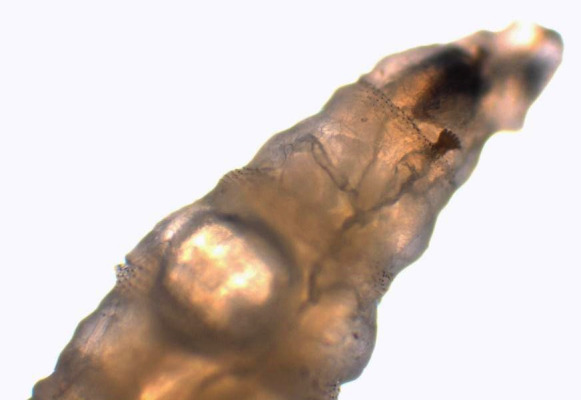
Larve ne présentant pas de capsule céphalique définie, sclérotique ou rigide. On note la présence des stigmates respiratoires antérieurs (grossissement x10) Larva without a definite, hard, sclerotized head capsule. Anterior spiracles are visible (magnification x10)

**Figure 3:**
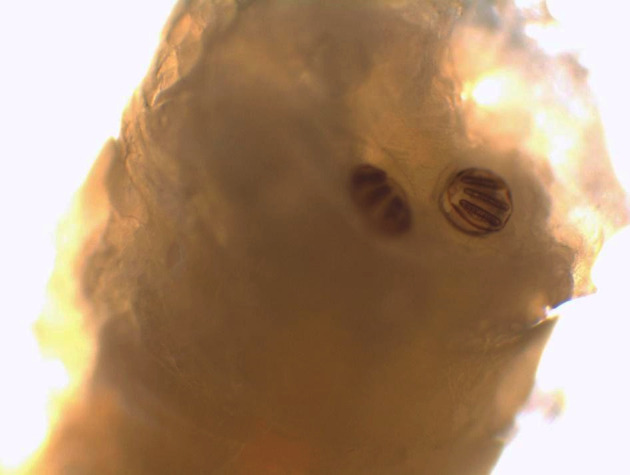
Spiracles respiratoires postérieurs avec un péritrème fermé et trois fentes droites convergeant vers le bouton bien visible non intégré dans le péritrème Posterior respiratory spiracles with closed peritreme stigma, three slits pointing to the distinct button not embedded in the peritreme

**Figure 4:**
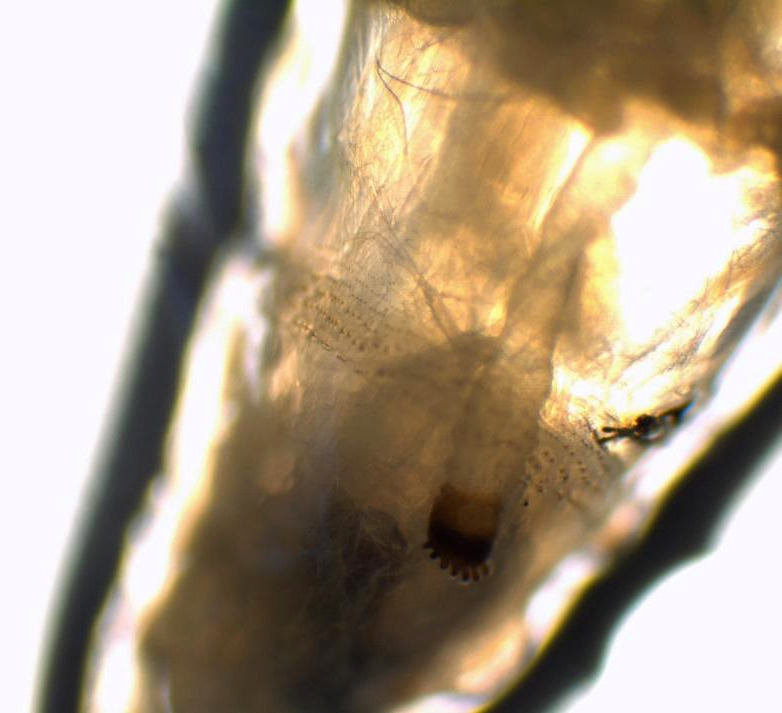
Les spiracles antérieurs sont formés de sept branches sur une seule rangée: aspect en doigts de gant. Aspect compatible avec le stade 3 des larves de *Lucilia* sp. *Anterior spiracles showing seven branches in a single row, aspect of immersion sleeve compatible with the third instar larva of* Lucilia *sp.*

Au laboratoire, nous avons reçu deux larves mobiles de 12 et 15 mm de longueur, à corps lisse, cylindrique, rétréci à l'avant et portant deux crochets ou sclérites buccaux à l'extrémité (Fig. 1). Des asticots sont fortement suspectés. Les larves ont été submergées dans l’éthanol à 70% avant leur examen microscopique. Nous avons utilisé un microscope optique au grossissement x4 puis x10. Les asticots sont portés par une lame porte-objet. Nous avons utilisé la clef de Ishijima [7] et « Pictorial key to some common species » de H.R. Dodge [6]. Les larves n'avaient pas de capsule céphalique définie, sclérotique ou rigide (Fig. 2). L'examen des spiracles respiratoires postérieurs a trouvé un péritrème fermé avec trois fentes droites convergeant vers un bouton bien visible non intégré dans le péritrème (Fig. 3). Les spiracles antérieurs sont formés de sept branches sur une seule rangée lui conférant un aspect en doigts de gant (Fig. 4). Cette description est compatible avec le stade 3 des larves de *Lucilia* sp.

Après la confirmation diagnostique de myiase et la bénignité présumée de ces larves, un lavage intense des cavités nasales par du sérum physiologique a été réalisé chez le patient, retirant quelques autres larves. Aucun asticot n'a été observé dans les jours suivant, mais malheureusement le patient est décédé suite aux complications du choc septique.

## Discussion

La myiase humaine est causée par des mouches volantes qui déposent leurs oeufs sur les tissus sains ou nécrosés, les plaies infectées et malodorantes ou occasionnellement dans les orifices nasaux, oraux ou auditifs [9]. Au Maroc, les myiases sont généralement causées par des espèces de la famille des Oestridae et spécifiquement *Oestrus ovis* [12]. Il existe quelques cas de myiases rapportés autour du monde chez des patients hospitalisés [1, 3, 9], les facteurs exposant à cette infection étant les plaies ouvertes, l'immobilisation des patients, les infections et les pathologies systémiques sous-jacentes ainsi que toutes les conditions causant une immunodépression des patients. Dans des unités de soins intensifs, les patients peuvent être accidentellement exposés à ce risque [2, 6, 11]. Cette situation, très rare, est causée par l'incapacité des patients intubés à repousser les mouches volantes. Les mouches sont souvent attirées par l'odeur des plaies infectées et la présence de sang, surtout dans un climat chaud et humide. Notre patient était en sédation, présentant une péritonite opérée et hospitalisé dans un milieu relativement humide et chaud formant ainsi un climat favorable. Tout cela a créé de bonnes conditions pour que les mouches assurent leur cycle de vie évolutif et donc la ponte des oeufs.

La plupart des myiases cavitaires sont causées par le genre *Lucilia* [9], de même que la majorité des cas rapportés aux unités de soins intensifs [2, 8]. *Lucilia* appartient à la famille des Calliphoridae. Ce sont des mouches de viande de taille moyenne qui pondent leurs oeufs sur les viandes fraîches ou cuites et parfois sur les tissus des animaux vivants. Le genre *Lucilia* rassemble des mouches avec un éclat vert métallique. Elles peuvent être utilisées dans le traitement des plaies par l'induction d'une myiase artificielle strictement contrôlée. Ceci est possible grâce à leur forte affinité aux tissus nécrosés alors que les tissus propres et sains ne les attirent pas. Toutefois, *Lucilia* peut causer accidentellement une myiase facultative [10].

Le diagnostic de la mouche responsable d'une myiase est d'importance indiscutable puisque toutes les myiases ne sont pas bénignes. Certains asticots peuvent causer chez les hôtes des lésions tissulaires graves conduisant, dans les cas extrêmes, à leur décès [5]. Le traitement des myiases reste le retrait de la totalité des larves manuellement ou par endoscopie ou chirurgie dans les infections profondes. L'utilisation de l'association de l'ivermectine, antihelminthique à large spectre, est possible selon les espèces incriminées et la situation clinique des patients [4].

La lutte contre les mouches en milieu hospitalier est indispensable dans la prévention de cette infection parasitaire. Elle est assurée par l’éviction du stockage des denrées alimentaires, la destruction rapide des déchets de soins, la protection des plaies par des pansements et les soins adéquats des infections. La mise en place d'un plan de désinsectisation est indispensable. Elle peut être chimique par des organophosphorés ou pyréthrinoïdes, ou mécanique par désinsectiseurs à UV, électriques ou à plaque de glu [3].

## Conclusion

Une myiase contractée à l'hôpital révèle un sérieux problème de soin. Il n'existe jusquelà aucun consensus pour leur traitement, mais l'extirpation de toutes les larves est la conduite la plus sûre et la plus efficace.

## Liens D'intérêts

Les auteurs ne déclarent aucun lien d'intérêt.

## Contribution des Auteurs

Manal Bouikhif: Rédaction du brouillon original, révision, édition et ressources.

Yasmine El Kettani: Ressources.

Mohamed Lyagoubi: Encadrement.

Sarra Aoufi: Administration et validation du projet.
